# Impact of the Distance From the Cavotricuspid Isthmus to the Right Coronary Artery on First‐Pass Conduction Block During Cryoablation for Atrial Flutter

**DOI:** 10.1002/joa3.70159

**Published:** 2025-07-31

**Authors:** Minoru Nodera, Takashi Kaneshiro, Sadahiro Murota, Shinya Yamada, Masayoshi Oikawa, Yasuchika Takeishi

**Affiliations:** ^1^ Department of Cardiovascular Medicine Fukushima Medical University Fukushima Japan; ^2^ Department of Arrhythmia and Cardiac Pacing Fukushima Medical University Fukushima Japan

**Keywords:** atrial flutter, cavotricuspid isthmus, cryoablation, first‐pass conduction block, right coronary artery

## Abstract

**Background:**

Cryoablation is an alternative to radiofrequency ablation for the treatment of cavotricuspid isthmus (CTI)‐dependent atrial flutter (AFL). However, the anatomical features that make achieving a CTI conduction block using cryoablation challenging remain unclear.

**Methods:**

This study included 100 consecutive patients who underwent CTI cryoablation for AFL. Patients were divided into two groups: the first‐pass group, in which first‐pass CTI conduction block was achieved (*n* = 72) and the non‐first‐pass group, in which it was not achieved (*n* = 28). We analyzed the anatomical features and the temperature changes of the catheter during the first sequential CTI cryoablation.

**Results:**

The distance from the CTI to the right coronary artery (RCA) in the first‐pass group was significantly longer than that in the non‐first‐pass group (*p* < 0.001). The time to reach nadir freezing temperature in the ventricular side of the CTI was significantly shorter in the first‐pass group than in the non‐first‐pass group (*p* < 0.001). The time to reach nadir freezing temperature at the ventricular side of the CTI correlated inversely with the distance from the CTI to the RCA (*R* = −0.410, *p* < 0.001). The distance from the CTI to the RCA was the only significant factor associated with achieving first‐pass CTI conduction block (odds ratio, 4.801, *p* < 0.001).

**Conclusions:**

The distance from the CTI to the RCA was significantly associated with achieving first‐pass CTI conduction block by cryoablation. The warming effect of the RCA blood flow might prevent the CTI conduction block during cryoablation.

## Graphical abstract

A shorter distance from the cavotricuspid isthmus to the right coronary artery reduces the success rate of achieving first‐pass conduction block during cryoablation for cavotricuspid isthmus‐dependent atrial flutter, likely due to the warming effect of coronary blood flow.

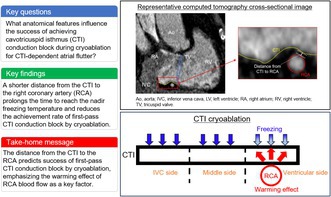

## 1 Introduction

Cavotricuspid isthmus (CTI)‐dependent atrial flutter (AFL) is a macroreentrant arrhythmia that circles counterclockwise around the tricuspid valve (TV). The CTI‐dependent AFL circuit involves the cavotricuspid isthmus (CTI), which is the target site for catheter ablation. Catheter ablation has been shown to be superior to antiarrhythmic drugs in the prevention of CTI‐dependent AFL recurrence and is currently used as first‐line therapy [1, 2, 3].

Recently, it has become possible to perform CTI cryoablation in addition to conventional radiofrequency (RF) ablation for CTI‐dependent AFL. Outcomes for CTI cryoablation have been reported to be comparable to those of CTI RF ablation [4, 5, 6]. Furthermore, compared to RF ablation, cryoablation has the advantages of being safer with a lower risk of myocardial perforation and blood clot formation and causing less pain to the patient [4, 5, 6].

Although most cases of CTI ablation achieve first‐pass conduction block, defined as creating a complete bidirectional conduction block of the CTI by first sequential ablation, there are some cases in which first‐pass conduction block is difficult to achieve. In CTI RF ablation, several anatomical factors have been proposed as influencing the difficulty of creating a conduction block of the CTI [7, 8, 9, 10]. Cryoablation differs from RF ablation in its lesion formation characteristics [11, 12], and therefore, the anatomical factors influencing CTI ablation that have been described for RF ablation may also have different effects on cryoablation. However, these differences remain unclear. In this study, we investigated the anatomical factors affecting the achievement of first‐pass conduction block of the CTI by cryoablation for CTI‐dependent AFL.

## 2 Methods

### 2.1 Study Population

This retrospective study included 100 consecutive patients (68 men; mean age, 65.9 ± 10.6 years) who underwent CTI cryoablation for CTI‐dependent AFL at Fukushima Medical University Hospital between April 2016 and May 2023 using an 8 mm tip cryocatheter. All patients also had atrial fibrillation (AF) and initially underwent pulmonary vein isolation (PVI) for AF using a cryoballoon followed by CTI cryoablation. A computed tomography (CT) scan with contrast injection was performed for anatomic evaluation before ablation in all the patients. The cases in which CT image evaluation was difficult due to artifacts were excluded. The study protocol was approved by the ethical committee of Fukushima Medical University, and written informed consent was obtained from all study participants.

### 2.2 Ablation Protocol

Surface and bipolar intracardiac electrograms (ECGs) were continuously recorded and stored on a computer‐based digital recording system. The procedure was performed under moderate sedation with dexmedetomidine. A 6Fr 10‐pole mapping catheter (CS Catheter with Auto ID Technology, Biosense Webster Inc., Diamond Bar, CA) was inserted through the right femoral vein into the coronary sinus. Following the achievement of PVI using a cryoballoon (Arctic Front Advance, Medtronic Inc., Minneapolis, MN), a 5Fr 10‐pole mapping catheter (EP‐star, Japan Lifeline Co. Ltd., Tokyo, Japan) was positioned around the TV. CTI cryoablation was performed with an 8‐mm tip cryocatheter (Freezor MAX, Medtronic Inc.) and 15‐Fr steerable sheath (Flexcath Advance, Medtronic Inc., Minneapolis, MN) using a sequential application technique going point‐by‐point from the TV to the inferior vena cava (IVC). Ablation was performed at a target temperature of −80°C, and each cryoenergy delivery lasted 150 s. Ablation was performed upon confirmation of the catheter tip position in two fluoroscopic directions, the right anterior oblique (RAO) view and the left anterior oblique view, to avoid gaps between points. Acute ablation success was defined as complete bi‐directional conduction block along the CTI line. The formation of bi‐directional block was confirmed by recording widely spaced double potentials along the entire ablation line during pacing from the coronary sinus ostium as well as by using a differential pacing technique [13]. Nevertheless, potential‐guided gap mapping using the same ablation catheter and additional ablation was performed if the complete bidirectional conduction block was not achieved during the first sequential ablation. All procedures in this study were performed by three electrophysiologists with extensive experience in CTI ablation, and a consistent cryoablation protocol was used throughout the study period.

Patients were divided into two groups: the first‐pass group, in which complete bidirectional conduction block of the CTI was achieved during the first sequential ablation, and the non‐first‐pass group, in which complete bidirectional conduction block was not achieved.

### 2.3 Cardiac Computed Tomography Imaging

We performed electrocardiogram‐gated CT for image before ablation. A 320‐row multidetector CT scanner (Aquilion ONE/GENESIS Edition, Canon Medical Systems Co. Ltd., Otawara, Japan), synchronized with the patient's heartbeat, was used. During the image acquisition, the patient was kept in the supine position with an expiratory breath‐hold. Contrast agent was injected at a rate of 10 mgI/kg/s. Scanning was performed at 120 kV and modulated mA (SD 20). Reconstruction was done with 1.0‐mm‐thick slices at 1.0‐mm interval.

**FIGURE 1:**
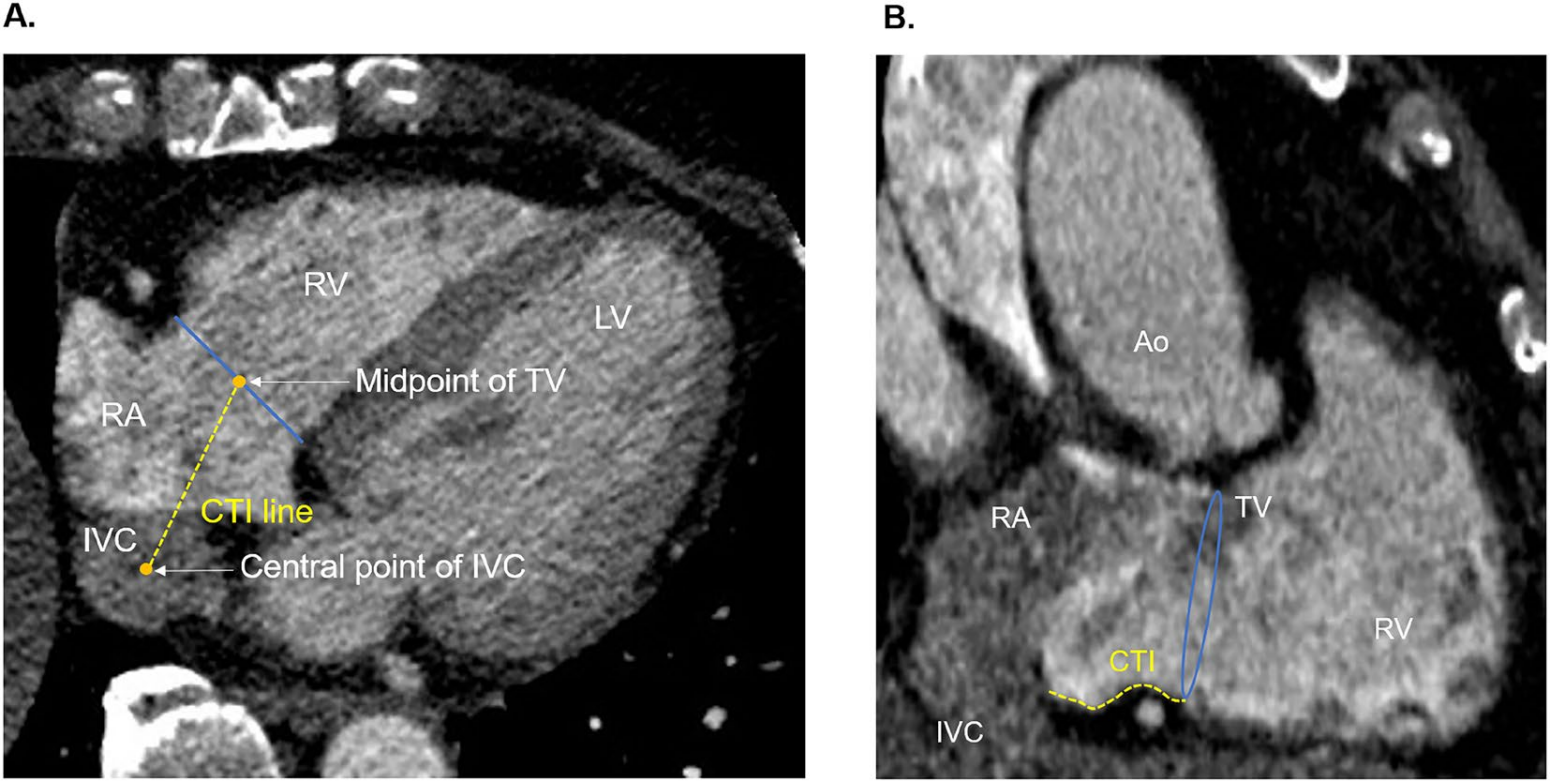
Representative computed tomography cross‐sectional images of the cavotricuspid isthmus (CTI) anatomical measurements. (A) A straight line connecting the central point of the inferior vena cava (IVC) and the midpoint of the tricuspid valve (TV) in the horizontal long‐axis view was defined as the CTI line. (B) CTI anatomy was evaluated in the vertical long‐axis view of the defined CTI line in the horizontal long‐axis view. Ao, aorta; LV, left ventricle; RA, right atrium; RCA, right coronary artery; RV, right ventricle.

**FIGURE 2:**
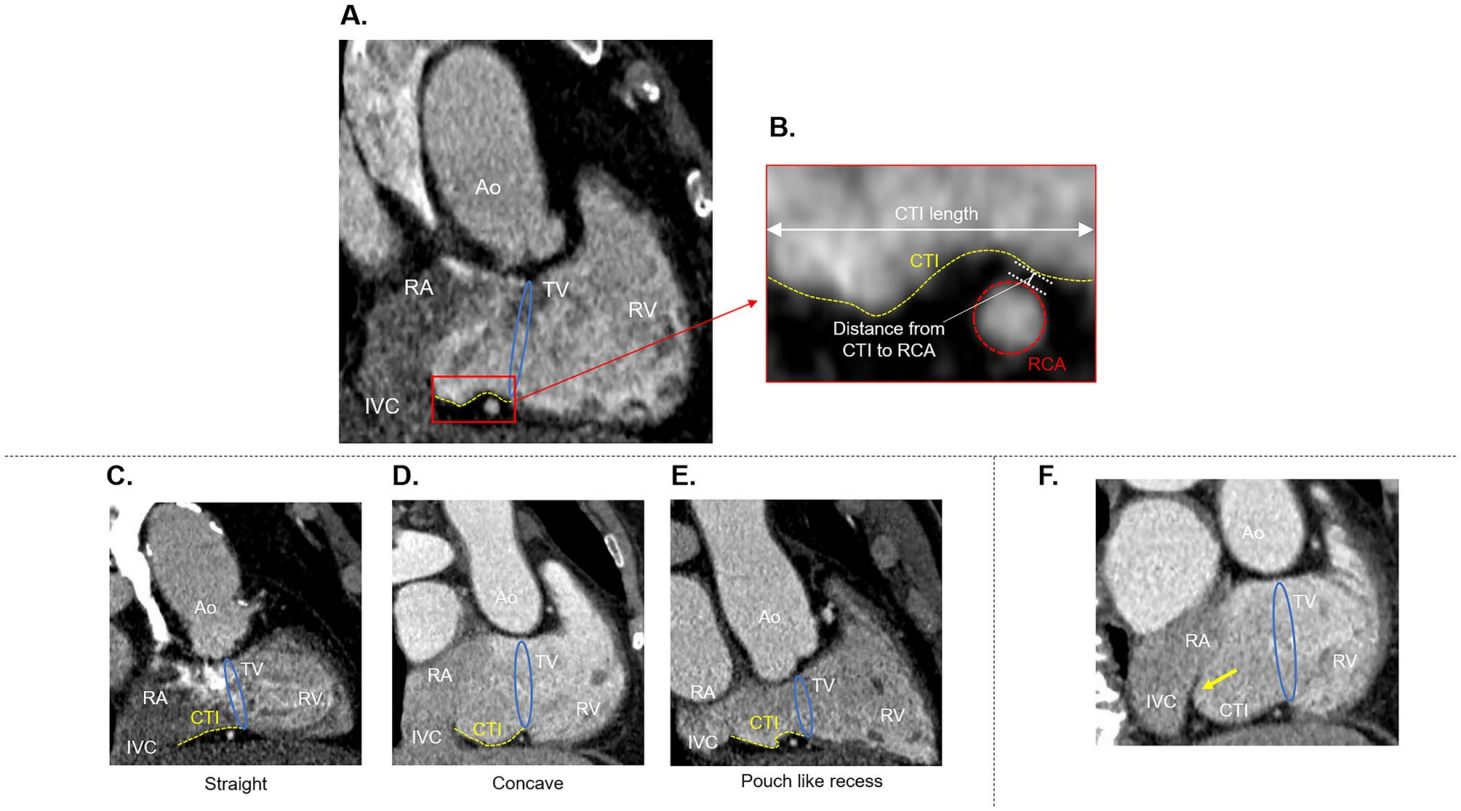
(A) Cavotricuspid isthmus (CTI) anatomy was assessed on the computed tomography images with the vertical long‐axis view of the defined CTI line in the horizontal long‐axis view. The endocardium of the CTI is indicated by the orange dashed line. (B) Measurements of the CTI length and the distance from the CTI to the right coronary artery (RCA). (C–E) Variants of CTI morphology evaluated using computed tomography were classified as straight (C), concave (D), and pouch‐like recess (E). (F) Yellow arrow indicates the presence of a prominent Eustachian valve at the junction of the IVC and CTI. Ao, aorta; LV, left ventricle; RA, right atrium; RV, right ventricle; TV, tricuspid valve.

Cardiologists, who were blinded to the patients' clinical information, manually evaluated the variable anatomical features of the CTI based on the CT data, using a computer workstation (Ziostation, Ziosoft Inc., Tokyo, Japan). For patients with AF at the time of the CT scan and insufficient quality of the end‐diastolic images, an end‐systolic data set was preferred. Based on a previous report [14], a straight line connecting the central point of the IVC and the midpoint of the TV in the horizontal long‐axis view was defined as the CTI line (Figure 1A). Next, CTI anatomy was evaluated in the vertical long‐axis view of this defined CTI line (Figures 1B and 2A), and the following items were measured.

The distance between the inferior hinge point of the TV and the junction of the IVC and the right atrium was defined as the length of CTI (Figure 2B).

The analysis of the CTI morphology was done as previously described (Figure 2C) [8]. The distance between the deepest point of the CTI and the line connecting the junction of the IVC and the right atrium and the inferior hinge point of the TV was measured. Straight morphology was defined as a distance ≤ 2 mm and concave morphology was defined as a distance > 2 mm. If the CTI could be divided into a pouch and flat vestibule, it was defined as pouch‐like recess morphology.

Presence of prominent Eustachian valve was defined as a Eustachian valve being 1‐mm thick and protruding for at least 10 mm in the right atrium from the border of the IVC (Figure 2D) [15].

The distance from the CTI to the right coronary artery was also analyzed (Figure 2B). Previous reports have divided the CTI equally into three regions: the ventricular side, middle side, and IVC side [16, 17, 18]. The right coronary artery (RCA) runs epicardially through the fat‐filled atrioventricular groove and is near the ventricular side of the CTI [10, 14, 16, 17, 18]. We measured the minimal distance from the endocardium of the ventricular side of the CTI to the adventitia of the RCA.

In addition, coronary artery anatomy was evaluated on the contrast‐enhanced CT images, and no patient showed significant stenosis or chronic total occlusion of the RCA.

### 2.4 Procedural Parameters of Cryoablation

**FIGURE 3:**
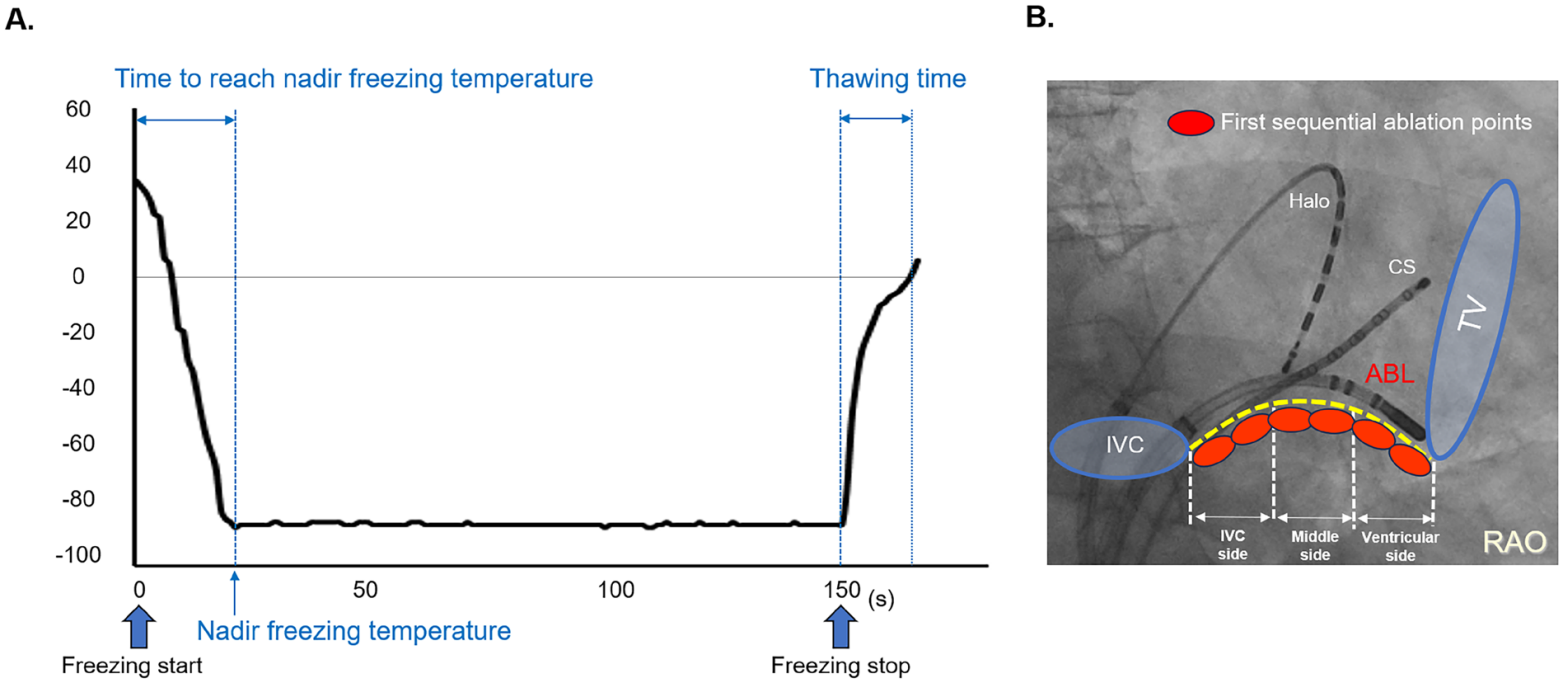
(A) Measuring the nadir freezing temperature, time to reach nadir freezing temperature, and thawing time as cryo‐application parameters. Thawing time was defined as the time between the end of the cryo‐application procedure and the temperature reaching 0°C at the catheter tip. (B) Representative right anterior oblique (RAO) fluoroscopic image of the three‐region classification of the cavotricuspid isthmus (CTI) first sequential ablation points. The averages for the nadir freezing temperature, time to reach nadir freezing temperature, and thawing time in each of the three regions were calculated. ABL, ablation catheter; CS, coronary sinus catheter; IVC, inferior vena cava; TV, tricuspid valve.

We evaluated the total freezing time in the first sequential ablation and the following parameters concerning the profiles of cryo‐applications: nadir freezing temperature, time to reach nadir freezing temperature, and thawing time (Figure 3A). Thawing time was defined as the time between the end of the cryo‐application procedure and the temperature reaching 0°C at the catheter tip. The CTI first sequential ablation sites were classified into the ventricular side, middle side, and IVC side on the fluoroscopic images of the RAO. The averages for the nadir freezing temperature, time to reach the nadir freezing temperature, and thawing time in each of the three regions were calculated (Figure 3B).

In addition, in the non‐first‐pass group, we analyzed where the final CTI conduction gap site was located within the three regions.

The procedure time was defined as the duration between the initiation of CTI ablation and the final confirmation of the CTI conduction block.

### 2.5 Follow Up for Recurrence

Post‐ablation follow‐up was conducted using regular clinical interviews along with 12‐lead and 24‐h Holter ECGs. We investigated not only the recurrence of CTI‐dependent AFL. Recurrence of CTI‐dependent AFL and was defined as a corresponding electrocardiographic recording lasting for more than 30 s after a blanking period of 3 months. Continuation or discontinuation of antiarrhythmic drug therapy after the procedure was determined by the primary physician.

### 2.6 Statistical Analysis

Continuous variables were expressed as mean ± standard deviation for normally distributed data, or as median and interquartile range for non‐normally distributed data. Normality was assessed using the Shapiro–Wilk test. Categorical variables were reported as percentages. Differences between the two groups were assessed using the unpaired *t*‐test or the Mann–Whitney *U*‐test for continuous variables, whereas the Kruskal–Wallis test was used to analyze differences for continuous variables among three groups. Categorical variables were assessed using Fisher's exact test.

To evaluate factors associated with first‐pass CTI conduction block, multivariate logistic regression analysis was performed using five variables selected based on clinical relevance and prior anatomical considerations: age, gender, CTI length, CTI morphology, and the distance from the CTI to the RCA. CTI morphology was further categorized as “simple” (straight morphology without a prominent Eustachian valve) and “complex” (concave morphology, pouch‐like recess, or presence of a prominent Eustachian valve).

A two‐sided *p* value < 0.05 was regarded as statistically significant. We used SPSS version 28 (IBM Corp., Armonk, NY) for the analyses.

## 3 Results

### 3.1 Baseline Patient Characteristics

The baseline patient characteristics are shown in Table 1. The mean age was 66 ± 11 years, and 68 of the 100 patients (68%) were male. Eighty‐seven of the 100 patients (87%) had paroxysmal AF. The median left atrial volume index and the median right atrial area measured by echocardiography were 40.0 [31.0–48.0] mL/m^2^ and 18.0 [13.0–23.0] cm^2^, respectively.

**TABLE 1:** Baseline patient characteristics.

	Total (*n* = 100)	First‐pass group (*n* = 72)	Non‐first‐pass group (*n* = 28)	*p*
Age, years	66 ± 11	66 ± 11	65 ± 11	0.482
Male, *n* (%)	68 (68)	50 (69)	18 (64)	0.620
Body mass index, kg/m^2^	23.6 ± 3.7	23.1 ± 3.3	25.0 ± 4.4	0.056
Paroxysmal atrial fibrillation, *n* (%)	87 (87)	61 (85)	26 (93)	0.277
Hypertension, *n* (%)	58 (58)	44 (61)	14 (50)	0.312
Diabetes, *n* (%)	10 (10)	7 (10)	3 (11)	0.882
Left atrial volume index (mL/m^2^)	40.0 [31.0–48.0]	39.0 [31.0–48.0]	42.0 [32.0–51.8]	0.494
Left ventricular ejection fraction, %	64.0 [59.0–69.0]	62.0 [56.7–69.0]	65.0 [60.0–68.8]	0.285
Right atrial area, cm^2^	18.0 [13.0–23.0]	18.5 [13.0–22.3]	17.5 [14.5–26.5]	0.361
Estimated GFR, mL/min/1.73 m^2^	61.7 ± 15.2	60.8 ± 15.1	64.0 ± 15.4	0.339
Brain natriuretic peptide, pg/mL	55.5 [19.5–124.6]	57.7 [17.6–129.9]	48.6 [15.3–225.8]	0.694

*Note:* Values are reported as the mean ± standard deviation, median [25th–75th percentile], or number of patients (%).

Abbreviation: GFR, glomerular filtration rate.

### 3.2 CTI Cryoablation Results

CTI cryoablation was successfully completed in all 100 patients. To further evaluate the potential impact of the learning curve and inter‐operator variability on procedural outcomes, we performed a post hoc comparison of the first‐pass CTI conduction block success rate and procedure time between the first 50 cases and the last 50 cases. The results showed no significant differences between the two groups in either the first‐pass conduction block rate (74% vs. 70%, *p* = 0.656) or the procedure time (24 [18–32] min vs. 26 [21–37] min, *p* = 0.104). Based on the ablation procedure results, 72 patients were classified into the first‐pass group, with the remaining 28 patients being classified into the non‐first‐pass group. There were no significant differences in the baseline characteristics between the two groups (Table 1). In the non‐first‐pass group, the final CTI conduction gap sites were on the ventricular side in 17 patients (61%), the middle side in four patients (14%), and the IVC side in seven patients (25%), with the ventricular side being the most common conduction gap site.

### 3.3 Comparison of Anatomical Features of CTI

The comparison of the anatomical features of the CTI between the two groups is shown in Table 2. There were no significant differences between the two groups in the CTI length, CTI morphology, and presence of a prominent Eustachian valve. On the other hand, the distance from the CTI to the RCA was significantly longer in the first‐pass group than in the non‐first‐pass group (3.3 ± 0.8 mm vs. 2.5 ± 0.7 mm, *p* < 0.001). In the non‐first‐pass group, we compared the distance from the CTI to the RCA for each of the three regions of the conduction gap site; however, no significant differences were observed (Table S1).

**TABLE 2:** Comparison of the anatomical features of cavotricuspid isthmus.

	First‐pass group (*n* = 72)	Non‐first‐pass group (*n* = 28)	*p*
CTI length (cm)	2.4 ± 0.6	2.6 ± 0.6	0.165
CTI morphology, *n* (%)			
Straight	36 (50)	12 (43)	0.521
Concave	23 (32)	9 (32)	0.985
Pouch‐like recess	13 (18)	7 (25)	0.436
Prominent Eustachian valve, *n* (%)	11 (15)	5 (18)	0.752
Distance from CTI to RCA (mm)	3.3 ± 0.8	2.5 ± 0.7	< 0.001

*Note:* Values are reported as the mean ± standard deviation or number of patients (%).

Abbreviations: CTI, cavotricuspid isthmus; RCA, right coronary artery.

### 3.4 Procedural Parameters During Cryoablation

The comparison of the procedural parameters during cryoablation between the two groups is shown in Table 3. There were no significant differences in the nadir freezing temperature and thawing time at any of the three regions between the two groups. The time to reach nadir freezing temperature at the ventricular side of the CTI was significantly shorter in the first‐pass than in the non‐first‐pass group (28.0 [25.8–33.3] sec vs. 30.0 [28.0–38.0] sec, *p* < 0.001), while there was no significant difference on the middle and IVC side of the CTI.

**TABLE 3:** Comparison of procedural parameters during cryoablation.

	First‐pass group (*n* = 72)	Non‐first‐pass group (*n* = 28)	*p*
Ventricular side of CTI			
Nadir freezing temperature (°C)	−82.5 [−84.0 to −81.0]	−83.0 [−84.0 to −82.0]	0.298
Time to reach nadir freezing temperature (s)	28.0 [25.8 to 33.3]	30.0 [28.0 to 38.0]	< 0.001
Thawing time (s)	8.0 [6.0 to 9.0]	8.0 [5.8 to 9.0]	0.886
Middle side of CTI			
Nadir freezing temperature (°C)	−84.0 [−85.0 to −83.0]	−84.0 [−85.5 to −82.8]	0.645
Time to reach nadir freezing temperature (s)	21.5 [19.8 to 24.0]	23.0 [20.5 to 25.0]	0.796
Thawing time (s)	8.0 [7.0 to 10.0]	9.5 [6.5 to 14.3]	0.198
IVC side of CTI			
Nadir freezing temperature (°C)	−84.0 [−85.3 to −83.0]	−84.0 [−87.0 to −82.0]	0.846
Time to reach nadir freezing temperature (s)	21.0 [19.0 to 23.0]	22.5 [20.8 to 26.3]	0.764
Thawing time (s)	9.0 [7.0 to 11.0]	9.5 [6.0 to 14.3]	0.985
Total freezing time in first line (times)	5.0 [4.0 to 5.0]	5.0 [4.0 to 6.0]	0.192
Procedure time (min)	23 [18 to 27]	38 [30 to 46]	< 0.001

*Note:* Values are reported as the mean ± standard deviation, median [25th–75th percentile], or number of patients (%).

Abbreviations: CTI, cavotricuspid isthmus; IVC, inferior vena cava.

Total freezing time in the first line showed no difference between the two groups. Procedure time was significantly shorter in the first‐pass group than in the non‐first‐pass group (23 [18−27] min vs. 38 [30–46] min, *p* < 0.001).

Table S1 shows a comparison of the freezing parameters for each of the three regions of the conduction gap in the non‐first‐pass group; nonetheless, no significant differences were observed in any of the groups.

### 3.5 Correlation Between Time to Reach Nadir Freezing Temperature on the Ventricular Side of CTI and Distance From CTI to RCA

**FIGURE 4:**
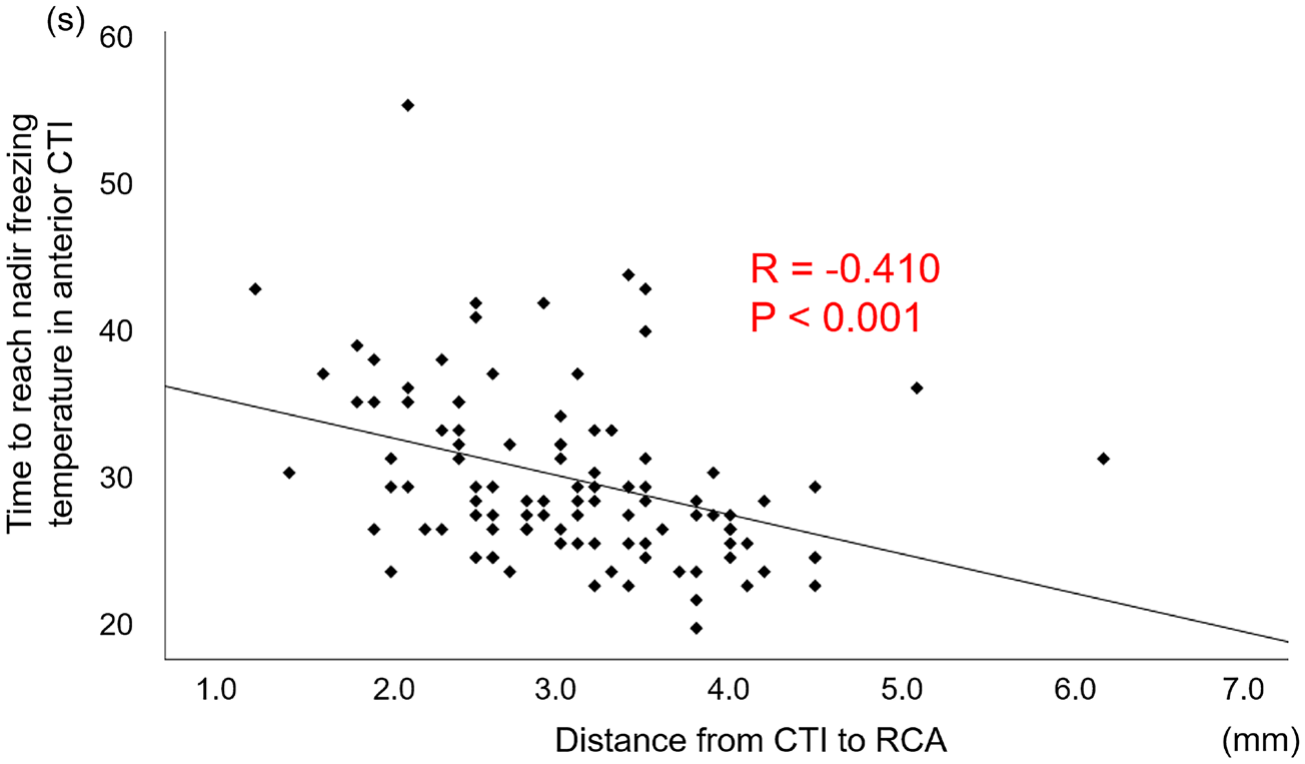
Correlation between the time to reach nadir freezing temperature on the ventricular side of the cavotricuspid isthmus (CTI) and the minimum distance from the CTI to the right coronary artery (RCA).

The correlation between the time to reach nadir freezing temperature on the ventricular side of the CTI and the distance from the CTI to the RCA is shown in Figure 4. A negative correlation was found between the time to reach nadir freezing temperature on the ventricular side of the CTI and the distance from the CTI to the RCA (*R* = −0.410, *p* < 0.001).

### 3.6 Factors Associated With First‐Pass Conduction Block of the CTI by Cryoablation

Multivariate logistic regression analysis was performed to assess factors associated with first‐pass conduction block of the CTI by cryoablation. The results are shown in Table 4. Among the evaluated variables, the distance from the CTI to the RCA was the only significant factor associated with achieving first‐pass conduction block (odds ratio, 4.801, 95% confidence interval 2.141–10.764, *p* < 0.001).

**TABLE 4:** Multivariate logistic regression analysis examining factors associated with first‐pass conduction block of the cavotricuspid isthmus (CTI) during cryoablation for CTI‐dependent atrial flutter.

	Odds ratio	95% confidence interval	*p*
Age (years)	1.024	0.976–1.075	0.330
Gender (male)	0.912	0.295–2.818	0.873
CTI length (cm)	0.488	0.219–1.088	0.080
Complex CTI morphology	0.616	0.217–1.746	0.362
Distance from CTI to RCA (mm)	4.801	2.141–10.764	< 0.001

Abbreviation: RCA, right coronary artery.

### 3.7 Recurrence Following Cryoablation

Recurrence of CTI‐dependent AFL was observed in eight patients during a mean follow‐up period of 18 ± 14 months, with six in the first‐pass group and two in the other group; however, the recurrence rates between the two groups did not significantly differ.

## 4 Discussion

The major findings of our study assessing CTI cryoablation for CTI‐dependent AFL were as follows: (1) the first‐pass group had a significantly longer distance between the CTI and the RCA than the non‐first‐pass group; (2) the time to reach nadir freezing temperature on the ventricular side of the CTI during CTI cryoablation was significantly shorter in the first‐pass group than in the non‐first‐pass group; (3) the time to reach nadir freezing temperature on the ventricular side of the CTI correlated inversely with the distance from the CTI to the RCA; and (4) the distance from the CTI to the RCA was the only significant factor associated with achieving first‐pass conduction block of CTI by cryoablation.

### 4.1 Proximity of RCA and CTI

Previous studies have shown that the RCA is closely located beneath the endocardium on the ventricular side of the CTI [10, 14, 16, 17, 18]. Using CT, Aloul et al. have reported that the mean minimal distance from the endocardium of the ventricular side in the central isthmus of the CTI to the adventitia of the RCA is 3 ± 2 mm [14], consistent with our results.

### 4.2 Warming Effect of RCA

In CTI RF ablation of CTI‐dependent AFL, the shorter the distance from the RCA to the CTI, the more difficult it is to create a CTI conduction block, requiring a high‐power setting for completion [10]. It has been proposed that the flow of blood at 37°C within the RCA has a convective cooling effect, protecting the surrounding atrial myocardial strands and potentially allowing for the recovery of conduction on the ventricular side of the CTI after ablation [10]. In our study, which examined CTI cryoablation, the proximity of the RCA to the CTI was also a factor related to first‐pass conduction block of the CTI. Moreover, in the non‐first‐pass group, the final conduction gap site was most commonly observed on the ventricular side of the CTI. These results suggest that RCA blood flow affected the lesion formation in the ventricular side of the CTI during cryoablation as well as RF ablation. The negative correlation observed between the distance from the CTI to the RCA and the time to reach nadir freezing temperature on the ventricular side of the CTI provides important insights into the underlying mechanism. This indicated that the shorter the distance from the CTI to the RCA, the longer the time to reach nadir freezing temperature on the ventricular side of the CTI. Therefore, the RCA blood flow may have a warming effect on the ventricular side of the CTI during cryoablation, and the closer the RCA to the endocardium, the stronger the warming effect. Similar observations have been reported in experimental studies. Aoyama et al. demonstrated in a canine model that cryoablation near coronary arteries led to smaller lesion formation, likely due to the protective effect of coronary blood flow [19]. Likewise, Skanes et al. reported the safety and feasibility of cryoablation near coronary arteries and hypothesized that local blood flow may attenuate cryothermal injury [20]. These findings reinforce our hypothesis that the RCA may act as a heat source during cryoablation, limiting lesion formation on the ventricular side of the CTI.

Furthermore, the stronger the warming effect of the RCA blood flow, the more likely it is that lesion formation on the ventricular side of the CTI is inhibited, and that the same area becomes a conduction gap, resulting in failure to achieve first‐pass conduction block. In the present study, a comparative analysis of the three gap sites in the non‐first‐pass group revealed that the distance from CTI to RCA or the freezing parameters did not significantly differ. However, with an increased number of cases, significant differences may emerge between the ventricular side and the other two regions.

### 4.3 Advantages of Cryoablation in Avoiding RCA Injury

RF ablation and cryoablation differ in their lesion formation characteristics. RF ablation produces lesions with indistinct and heterogeneous boundaries relative to normal tissue, whereas cryoablation results in lesions with distinct and homogeneous boundaries [11, 12]. These differences arise from the varying mechanisms of tissue injury between the two modalities [11, 12]. Accordingly, the impact of RCA blood flow on lesion formation may differ between RF and cryoablation.

One significant advantage of cryoablation is its ability to avoid severe RCA injury, such as irreversible stenosis or occlusion [21, 22]. RF CTI ablation has been associated with cases of irreversible RCA stenosis and occlusion requiring angioplasty [23, 24]. However, most cases of RCA stenosis or occlusion following cryoablation are transient and attributable to reversible coronary artery spasm [21, 22]. This benefit is likely due to the fact that cryoablation does not cause damage to the coronary artery endothelium [11, 12]. In this study as well, no cases of RCA injury were observed. These findings suggest that cryoablation can be performed safely without causing RCA injury, even in cases where the RCA is in close proximity to the CTI endocardium.

### 4.4 Relationship Between CTI Cryoablation and Other Anatomical Factors of CTI

There have been reports of factors that make CTI RF ablation difficult, such as paucity, concave shapes, and the presence of a prominent Eustachian valve [7, 8, 9, 10]. However, in this study, CTI morphology and the presence of a prominent EV were not predictive factors for first‐pass conduction block. Saygi et al. have also shown that anatomical morphology does not affect the acute success rate in CTI cryoablation [25]. This is thought to be because the catheter tip adheres to the tissue during cryoablation, allowing for stable cooling [25, 26]. It has also been reported that CTI length affects the acute success rate for both RF and cryoablation. In our study, CTI length was not found to be associated with first‐pass conduction block, possibly because the CTI length in our study was shorter than that in previous reports.

### 4.5 Clinical Implications

Achieving first‐pass conduction block of the CTI can reduce procedure time, in turn leading to a reduction in fluoroscopy dose and complications. Based on the results of our study, to achieve first‐pass conduction block of the CTI in cases where the RCA is close to the CTI endocardium, it may be necessary to create a larger lesion on the ventricular side of the CTI, considering the warming effect of the RCA blood flow.

Miyazaki et al. have investigated lesion formation in cryoablation with Freezor max in animal studies [27]. They found that it was possible to enlarge lesions by (1) putting the catheter in contact with the tissue in a more perpendicular direction; and (2) increasing the catheter contact force [27]. In RF ablation, it has been reported that using an increased catheter contact force and power enlarges the lesion, albeit with an increased risk of the steam pop phenomenon, which causes myocardial rupture from a rapid rise in tissue temperature [28, 29]. In CTI RF ablation, first‐pass conduction block rates of nearly 90% have been achieved by increasing the power and contact force; however, the rate of the steam pop phenomenon increases accordingly [30, 31]. On the other hand, cryoablation does not cause the steam pop phenomenon, so larger lesions can be obtained safely, even when the RCA is in close proximity to the CTI, which may lead to improved success rates of first‐pass conduction block of the CTI.

Routine preprocedural CT scans are rarely performed for standalone CTI ablation in CTI‐dependent AFL, likely due to concerns such as radiation exposure; however, it is frequently used in cases involving concurrent AF ablation. This is primarily for the anatomical evaluation of the left atrium and pulmonary veins, as well as thrombus exclusion. Given the frequent coexistence of AFL and AF [32], preprocedural CT imaging can provide valuable anatomical information that may aid in optimizing procedural strategies. The findings of our study reveal that CT imaging, when already performed for AF ablation, can provide predictive insights into the success of CTI cryoablation by assessing the distance from the CTI to the RCA. This anatomical evaluation may contribute to reducing procedure time and enhancing safety in these combined procedures, without introducing additional radiation exposure. Therefore, regarding the dual ablation procedures, incorporating these anatomical parameters into procedural planning could be a practical and beneficial approach.

### 4.6 Study Limitations

This study had some limitations. First, this was a retrospective study performed at one center with a limited number of patients. For example, anatomical factors such as the CTI length and morphology, which are known to influence procedural demands during CTI ablation, may not have been conclusively evaluated in this study owing to the relatively low number of observations. In addition, it has been reported that the first‐pass conduction block of the CTI achieved with RF ablation is associated with a lower recurrence rate of CTI‐dependent AFL [30]. However, in this study, no significant difference in the recurrence rate of CTI‐dependent AFL was observed between the first‐pass group and the non‐first‐pass group. Further studies with larger sample sizes are needed to clarify these issues in cryoablation therapy. Second, unlike RF ablation, cryoablation does not allow visualization of catheter tip orientation and contact force, so it was not possible to investigate these factors. Third, although we defined the CTI anatomically using contrast‐enhanced CT based on established methods [14], the actual ablation line during the procedure may not correspond precisely to the CT‐defined segment. This potential mismatch represents a critical methodological limitation, as the anatomical measurements serve as the basis for correlating with procedural outcomes. Future studies incorporating intra‐procedural imaging modalities such as intracardiac echocardiography or electroanatomical mapping systems may allow for more accurate localization and improve anatomical–functional correlation. Finally, although all procedures were performed by experienced electrophysiologists using a consistent protocol, and no significant differences in first‐pass conduction block rate or procedure time were observed between first and late cases, the potential influence of operator experience or procedural evolution over the 7‐year study period cannot be entirely excluded.

## 5 Conclusions

In conclusion, during cryoablation for CTI‐dependent AFL, the warming effect of the RCA may potentially inhibit lesion formation on the ventricular side of the CTI. Cryoablation is expected to safely and reliably form a conduction block of the CTI even in cases where the RCA is near the endocardium of the CTI on preoperative CT analysis.

## Ethics Statement

The study protocol was approved by the ethics committee of Fukushima Medical University.

## Consent

Written informed consent was obtained from all study subjects.

## Conflicts of Interest

Shinya Yamada is affiliated with the Department of Arrhythmia and Cardiac Pacing, which receives support from Abbott Medical Japan Co. Ltd., Biotronik Japan Inc., and Japan Lifeline Co. Ltd. These companies had no involvement in the design, conduct, or content of this study.

## Supporting information


Table S1.

## Data Availability

The data that support the findings of this study are available from the corresponding author upon reasonable request.

## References

[1] A. Natale , K. H. Newby , E. Pisanó , et al., “Prospective Randomized Comparison of Antiarrhythmic Therapy Versus First‐Line Radiofrequency Ablation in Patients With Atrial Flutter,” Journal of the American College of Cardiology 35 (2000): 1898–1904. 10841241 10.1016/s0735-1097(00)00635-5

[2] H. Bastani , N. Drca , P. Insulander , et al., “Cryothermal vs. Radiofrequency Ablation as Atrial Flutter Therapy: A Randomized Comparison,” Europace 15 (2013): 420–428. 22927662 10.1093/europace/eus261

[3] H. Poty , N. Saoudi , M. Nair , F. Anselme , and B. Letac , “Radiofrequency Catheter Ablation of Atrial Flutter. Further Insights Into the Various Types of Isthmus Block: Application to Ablation During Sinus Rhythm,” Circulation 94 (1996): 3204–3213. 8989130 10.1161/01.cir.94.12.3204

[4] H. Bastani , T. Bourke , F. Braunschweig , et al., “Cryoablation as Standard Treatment of Atrial Flutter: A Prospective, 2‐Center Study (CASTAF),” Acta Cardiologica 76 (2021): 267–271. 32208915 10.1080/00015385.2020.1721717

[5] S. Miyazaki , J. Iwasawa , H. Taniguchi , et al., “Creating Bidirectional Conduction Block in the Cavotricuspid Isthmus by Cryothermal Ablation With a Short Freeze Time: Insight From the Results With a 2‐Minute Freeze Cycle,” International Journal of Cardiology 224 (2016): 149–154. 27657464 10.1016/j.ijcard.2016.09.064

[6] M. Kuniss , K. Kurzidim , H. Greiss , et al., “Acute Success and Persistence of Bidirectional Conduction Block in the Cavotricuspid Isthmus One Month Post Cryocatheter Ablation of Common Atrial Flutter,” Pacing and Clinical Electrophysiology 29 (2006): 146–152. 16492299 10.1111/j.1540-8159.2006.00308.x

[7] H. Heidbüchel , R. Willems , H. van Rensburg , J. Adams , H. Ector , and F. Van de Werf , “Right Atrial Angiographic Evaluation of the Posterior Isthmus: Relevance for Ablation of Typical Atrial Flutter,” Circulation 101 (2000): 2178–2184. 10801759 10.1161/01.cir.101.18.2178

[8] A. Da Costa , E. Faure , J. Thévenin , et al., “Effect of Isthmus Anatomy and Ablation Catheter on Radiofrequency Catheter Ablation of the Cavotricuspid Isthmus,” Circulation 110 (2004): 1030–1035. 15326078 10.1161/01.CIR.0000139845.40818.75

[9] P. Kirchhof , M. Ozgün , S. Zellerhoff , et al., “Diastolic Isthmus Length and ‘Vertical’ Isthmus Angulation Identify Patients With Difficult Catheter Ablation of Typical Atrial Flutter: A Pre‐Procedural MRI Study,” Europace 11 (2009): 42–47. 19029130 10.1093/europace/eun308

[10] H. U. Klemm , T. F. Weber , C. Johnsen , P. G. Begemann , T. Meinertz , and R. Ventura , “Anatomical Variations of the Right Coronary Artery May Be a Source of Difficult Block and Conduction Recurrence in Catheter Ablation of CTI‐Dependent Atrial Flutter,” Europace 12 (2010): 1608–1615. 20823041 10.1093/europace/euq320

[11] P. Khairy , P. Chauvet , J. Lehmann , et al., “Lower Incidence of Thrombus Formation With Cryoenergy Versus Radiofrequency Catheter Ablation,” Circulation 107 (2003): 2045–2050. 12668527 10.1161/01.CIR.0000058706.82623.A1

[12] P. Khairy and M. Dubuc , “Transcatheter Cryoablation Part I: Preclinical Experience,” Pacing and Clinical Electrophysiology 31 (2008): 112–120. 18181919 10.1111/j.1540-8159.2007.00934.x

[13] D. Shah , M. Haïssaguerre , A. Takahashi , P. Jaïs , M. Hocini , and J. Clémenty , “Differential Pacing for Distinguishing Block From Persistent Conduction Through an Ablation Line,” Circulation 102 (2000): 1517–1522. 11004142 10.1161/01.cir.102.13.1517

[14] B. Al Aloul , G. Sigurdsson , I. Can , J. M. Li , R. Dykoski , and V. N. Tholakanahalli , “Proximity of Right Coronary Artery to Cavotricuspid Isthmus as Determined by Computed Tomography,” Pacing and Clinical Electrophysiology 33 (2010): 1319–1323. 20663073 10.1111/j.1540-8159.2010.02844.x

[15] T. A. Vale , J. D. Newton , E. Orchard , R. Bhindi , N. Wilson , and O. J. Ormerod , “Prominence of the Eustachian Valve in Paradoxical Embolism,” European Journal of Echocardiography 12 (2011): 33–36. 20813791 10.1093/ejechocard/jeq100

[16] J. A. Cabrera , D. Sánchez‐Quintana , J. Farré , J. M. Rubio , and S. Y. Ho , “The Inferior Right Atrial Isthmus: Further Architectural Insights for Current and Coming Ablation Technologies,” Journal of Cardiovascular Electrophysiology 16 (2005): 402–408. 15828885 10.1046/j.1540-8167.2005.40709.x

[17] W. Klimek‐Piotrowska , M. K. Hołda , M. Koziej , et al., “Clinical Anatomy of the Cavotricuspid Isthmus and Terminal Crest,” PLoS One 11 (2016): e0163383. 27682030 10.1371/journal.pone.0163383 PMC5040420

[18] J. B. Morton , P. Sanders , N. C. Davidson , P. B. Sparks , J. K. Vohra , and J. M. Kalman , “Phased‐Array Intracardiac Echocardiography for Defining Cavotricuspid Isthmus Anatomy During Radiofrequency Ablation of Typical Atrial Flutter,” Journal of Cardiovascular Electrophysiology 14 (2003): 591–597. 12875419 10.1046/j.1540-8167.2003.02152.x

[19] H. Aoyama , H. Nakagawa , J. V. Pitha , et al., “Comparison of Cryothermia and Radiofrequency Current in Safety and Efficacy of Catheter Ablation Within the Canine Coronary Sinus Close to the Left Circumflex Coronary Artery,” Journal of Cardiovascular Electrophysiology 16 (2005): 1218–1226. 16302908 10.1111/j.1540-8167.2005.50126.x

[20] A. C. Skanes , D. L. Jones , P. Teefy , et al., “Safety and Feasibility of Cryothermal Ablation Within the Mid‐ and Distal Coronary Sinus,” Journal of Cardiovascular Electrophysiology 15 (2004): 1319–1323. 15574185 10.1046/j.1540-8167.2004.04116.x

[21] B. I. Johansson , T. J. Hrafnkelsdóttir , and N. Edvardsson , “ST Segment Elevation and Chest Pain During Cryoablation of Atrial Flutter,” Europace 9 (2007): 407–410. 17440004 10.1093/europace/eum046

[22] N. V. Pothineni , K. Kancharla , A. J. Katoor , et al., “Coronary Artery Injury Related to Catheter Ablation of Cardiac Arrhythmias: A Systematic Review,” Journal of Cardiovascular Electrophysiology 30 (2019): 92–101. 30288838 10.1111/jce.13764

[23] S. Ouali , F. Anselme , A. Savouré , and A. Cribier , “Acute Coronary Occlusion During Radiofrequency Catheter Ablation of Typical Atrial Flutter,” Journal of Cardiovascular Electrophysiology 13 (2002): 1047–1049. 12435195 10.1046/j.1540-8167.2002.01047.x

[24] N. Raio , T. J. Cohen , R. Daggubati , and K. Marzo , “Acute Right Coronary Artery Occlusion Following Radiofrequency Catheter Ablation of Atrial Flutter,” Journal of Invasive Cardiology 17 (2005): 92–93. 15687532

[25] S. Saygi , H. Bastani , N. Drca , et al., “Impact of Cavotricuspid Isthmus Morphology in CRYO Versus Radiofrequency Ablation of Typical Atrial Flutter,” Scandinavian Cardiovascular Journal 51 (2017): 69–73. 27826985 10.1080/14017431.2016.1259496

[26] A. C. Skanes , G. Klein , A. Krahn , and R. Yee , “Cryoablation: Potentials and Pitfalls,” Journal of Cardiovascular Electrophysiology 15, no. Supplement (2004): S28–S34. 15482458 10.1046/j.1540-8167.2004.15106.x

[27] S. Miyazaki , H. O'Connell , and B. Maus , “Parameters Associated With Acute Morphometric Lesion Dimensions Created by Cryocatheters,” Journal of Interventional Cardiac Electrophysiology 54 (2019): 109–118. 30251226 10.1007/s10840-018-0452-x

[28] A. Ikeda , H. Nakagawa , H. Lambert , et al., “Relationship Between Catheter Contact Force and Radiofrequency Lesion Size and Incidence of Steam Pop in the Beating Canine Heart: Electrogram Amplitude, Impedance, and Electrode Temperature Are Poor Predictors of Electrode‐Tissue Contact Force and Lesion Size,” Circulation. Arrhythmia and Electrophysiology 7 (2014): 1174–1180. 25381331 10.1161/CIRCEP.113.001094

[29] Y. M. Hwang , W. S. Lee , K. J. Choi , and Y. R. Kim , “Radiofrequency Induced Lesion Characteristics According to Force‐Time Integral in Experimental Model,” Medicine 100 (2021): e25126. 33725912 10.1097/MD.0000000000025126 PMC7969321

[30] G. Viola , G. Stabile , S. Bandino , et al., “Safety, Efficacy, and Reproducibility of Cavotricuspid Isthmus Ablation Guided by the Ablation Index: Acute Results of the FLAI Study,” Europace 23 (2021): 264–270. 33212484 10.1093/europace/euaa215

[31] A. Chikata , T. Kato , K. Usuda , et al., “Ablation Index‐Guided High‐Power vs. Moderate‐Power Cavotricuspid Isthmus Ablation,” Heart and Vessels 38 (2023): 90–95. 35852611 10.1007/s00380-022-02125-9

[32] X. J. Dong , B. B. Wang , F. F. Hou , et al., “Global Burden of Atrialfibrillation/Atrial Flutter and Its Attributable Risk Factors From 1990 to 2019,” Europace 25 (2023): 793–803. 36603845 10.1093/europace/euac237 PMC10062373

