# Supplementary material for: Impact of the Distance From the Cavotricuspid Isthmus to the Right Coronary Artery on First‐Pass Conduction Block During Cryoablation for Atrial Flutter
Source: J Arrhythm. 2025 Jul 31;41(4):e70159. doi: 10.1002/joa3.70159 (PMC12314311; doi:10.1002/joa3.70159)
Supplement: Supplementary file 1 — Table S1. [file JOA3-41-e70159-s001.docx]

**Supplemental Table 1. Comparison of the distance from cavotricuspid isthmus to right coronary artery and freezing parameters in the three regions of the conduction gap**

|  | **Conduction gap in Ventricular side**  **(n = 17)** | **Conduction gap in Middle side**  **(n = 4)** | **Conduction gap in IVC side**  **(n = 7)** | ***P* value** |
| --- | --- | --- | --- | --- |
| Distance from RCA to CTI (cm) | 2.2 [1.9−3.1] | 3.2 [2.4−3.4] | 2.5 [2.1− 2.6] | 0.150 |
|  |  |  |  |  |
| **Ventricular side of CTI** |  |  |  |  |
| Nadir freezing temperature (°C) | -83.0 [-83.8− -81.5] | -83.0 [-83.8− -82.3] | -83.0 [-84.0− -81.0] | 0.583 |
| Time to reach nadir freezing temperature (s) | 32.0 [29.0−38.0] | 39.0 [31.8−43.3] | 27.0 [25.0−30.0] | 0.127 |
| Thawing time (s) | 6.0 [5.0−9.0] | 7.5 [4.8−8.8] | 9.0 [8.0−16.0] | 0.964 |
| **Middle side of CTI** |  |  |  |  |
| Nadir freezing temperature (°C) | -84.0 [-85.0− -83.0] | -85.0 [-86.5− -84.3] | -84.0 [-85.0− -82.0] | 0.145 |
| Time to reach nadir freezing temperature (s) | 23.0 [20.0−24.0] | 25.0 [20.5−26.5] | 21.0 [20.0−23.0] | 0.174 |
| Thawing time (s) | 10.0 [7.5−15.5] | 8.0 [5.8-16.3] | 9.0 [6.0−11.0] | 0.588 |
| **IVC side of CTI** |  |  |  |  |
| Nadir freezing temperature (°C) | -84.0 [-86.0− -83.0] | -85.0 [-86.5− -84.3] | -85.5 [-86.0− -82.0] | 0.202 |
| Time to reach nadir freezing temperature (s) | 22.0 [19.0−24.5] | 22.5 [16.8−26.8] | 21.0 [18.0-26.0] | 0.893 |
| Thawing time (s) | 9.0 [6.0−13.0] | 11.0 [6.8−17.5] | 9.0 [7.0−11.0] | 0.528 |

CTI: cavotricuspid isthmus, IVC: inferior vena cava, RCA: right coronary artery.
